# COVID-19 Related Psychological Distress, Fear and Coping: Identification of High-Risk Groups in Bangladesh

**DOI:** 10.3389/fpsyt.2021.718654

**Published:** 2021-08-13

**Authors:** Muhammad Aziz Rahman, Shaila Rahman, Amit Wazib, S. M. Yasir Arafat, Zulfia Zinat Chowdhury, Bhuiyan Mohammad Mahtab Uddin, Mufti Munsurar Rahman, Ahmed Suparno Bahar Moni, Sheikh M. Alif, Farhana Sultana, Masudus Salehin, Sheikh Mohammed Shariful Islam, Wendy Cross, Tamanna Bahar

**Affiliations:** ^1^School of Health, Federation University Australia, Berwick, VIC, Australia; ^2^Australian Institute for Primary Care and Ageing, La Trobe University, Melbourne, VIC, Australia; ^3^Department of Noncommunicable Diseases, Bangladesh University of Health Sciences, Dhaka, Bangladesh; ^4^Faculty of Public Health, Universitas Airlangga, Surabaya, Indonesia; ^5^Enam Medical College and Hospital, Savar, Bangladesh; ^6^National Institute of Cancer Research and Hospital, Dhaka, Bangladesh; ^7^Advanced Medical and Dental Institute, Universiti Sains Malaysia, Penang, Malaysia; ^8^School of Public Health and Preventive Medicine, Monash University, Melbourne, VIC, Australia; ^9^Telstra Health, Melbourne, VIC, Australia; ^10^Melbourne School of Population and Global Health, University of Melbourne, Carlton, VIC, Australia; ^11^Institute for Physical Activity and Nutrition, Deakin University, Burwood, VIC, Australia

**Keywords:** COVID-19, mental health, psychological distress, coping, resilience, Bangladesh

## Abstract

**Background:** The COVID-19 pandemic has imposed psychological distress and fear across the globe; however, factors associated with those issues or the ways people cope may vary by country or context. This study aimed to investigate the factors associated with psychological distress, fear, and coping strategies for people living in Bangladesh during the COVID-19 pandemic.

**Methods:** A cross-sectional study conducted in August-September 2020 using online platforms in Bangladesh. People residing in Bangladesh, aged ≥18 years, who were proficient in English and able to respond to online questionnaire. The Kessler Psychological Distress Scale was used to assess the psychological stress. Level of fear was assessed using the Fear of COVID-19 Scale, and strategies to cope were assessed using the Brief Resilient Coping Scale.

**Results:** Of the 962 participants, half of them were aged between 30 and 59 years. Being born in Bangladesh, having graduate education, perceived distress due to employment change, effect of COVID-19 on financial situation, having multiple comorbidities, and visiting a healthcare provider in the last 4 weeks were associated with higher levels of both psychological distress and fear of COVID-19. Furthermore, higher psychological distress was associated with being a female (AOR 1.81, 95% CI 1.33–2.47, *p* < 0.001), being a frontline worker (AOR 1.50, 95% CI 1.04–2.15, *p* < 0.05), having pre-existing psychiatric problems (AOR 4.03, 95% CI 1.19–13.7, *p* < 0.05), being a smoker (AOR 2.02, 95% CI 1.32–3.09, *p* < 0.01), providing care to a known/suspected COVID-19 patient (AOR 1.96, 95% CI 1.40–2.72, *p* < 0.001), having a recent overseas travel history and being in self-quarantine (AOR 4.59, 95% CI 1.23–17.2, *p* < 0.05), self-isolation without COVID-19 (AOR 2.63, 95% CI 1.68–4.13, *p* < 0.001) or being COVID-19 positive (AOR 2.53, 95% CI 1.19–5.34, *p* < 0.05), and having high levels of fear of COVID-19 (AOR 3.27, 95% CI 2.29–4.66, *p* < 0.001). A higher level of fear was associated with moderate to high levels of psychological distress (AOR 3.29, 95% CI 2.31–4.69, *p* < 0.001). People with pre-existing mental health problems were less likely to be resilient (AOR 0.25, 95% CI 0.11–0.54, *p* < 0.01), whereas those with having an income were more likely to be resilient (AOR 1.46, 95% CI 1.02–2.11, *p* < 0.05).

**Conclusion:** Effective interventions to support the vulnerable groups including improved access to mental health services are of utmost importance during the pandemic.

## Introduction

The COVID-19 pandemic has affected more than 218 countries and territories across the world. Globally more than 140 million cases of COVID-19 and nearly 3 million deaths due to COVID 19 have been reported to date (1). The United States of America has reported the highest number of cases and deaths due to COVID-19 followed by India, Brazil, France, Russian Federation, the United Kingdom, Turkey, Italy and Spain. In Bangladesh, the first three cases of COVID-19 were detected on 8th March 2020. To date, around 715,252 confirmed COVID-19 cases and 10,283 deaths have been reported in Bangladesh (2). The Government of Peoples Republic of Bangladesh has developed a Multisectoral Action Plan in response to the COVID-19 pandemic preparedness. This includes lockdown in major cities, practizing of social distancing, closing of schools and universities, working from home arrangements where possible, widespread awareness campaigns for handwashing practices, complementary use of masks in public places including when using public transport, imposing regulation on international travel from hotspots, establishing quarantine centers, mandatory quarantine for COVID-19 suspected cases, and isolation of confirmed cases. Moreover, there are guidelines for COVID-19 clinical management, designation of public and private hospitals for treating positive cases, establishing isolation units in different hospitals, nationwide testing facilities, dissemination through health bulletins and tracking of COVID-19 infections and deaths through published data (3, 4).

Nationwide vaccination program has started in Bangladesh from February 7, 2021 (5). Despite all the Government efforts, Bangladesh had been experiencing the second wave of infection, started from March 1, 2021 (2). The strict restrictions on daily activities, social life and travel, the livelihood of the general population had been severely affected. Daily wage-earners have been affected most because of the restrictions on businesses, movement and public activities. Despite having a low case fatality rate (1.43%) compared to other countries, people are generally anxious and distressed due to the increasing number of new cases in the community and fear of death of those near and dear (4, 5).

During the early days of the pandemic, many frontline healthcare workers including emergency service providers such as police, armed forces personnel, bankers, and government officials were infected with COVID-19 (6). The scarcity of personal protective equipment, lack of an evidence-based treatment protocol, scarcity of resources made healthcare workers worried. Furthermore, healthcare workers were humiliated for providing care to COVID-19 and were asked to isolate and stay away from the community and families to curb the further spread of infection. This imposed enormous psychological distress and fear amongst the health workforce (7).

Several previous studies have found evidence of anxiety, depression, fear, sleep deprivation, and self-harm among community members during the pandemic (8). Studies also have shown that the COVID-19 pandemic affected people in different countries in different ways with some groups being more vulnerable than others. A recent review found that women, younger individuals, those living in rural areas, those with lower socioeconomic status, those are at high-risk of COVID-19 infection due to their work or high risk of having severe infection due to presence of comorbidities are associated with higher levels of anxiety and depression (9). In an Australian study, pre-existing mental health conditions, increased smoking and alcohol consumption during the locked down period, being female were associated with higher levels of psychological distress (10). In an Italian study, female gender, detachment with the friends and families were associated with higher levels of anxiety and stress (11). A recent study in China reported moderate to severe anxiety symptoms, distress, and depressive symptoms among community people (12). Few studies have been conducted in Bangladesh to assess the extent of mental health status during COVID-19 pandemic in different populations using different tools and methodologies. A study by Zubayer et al. showed moderate to extremely severe levels of depression, anxiety, and stress in the general population (4). Another study by Islam et al. showed a high prevalence of panic (79.6%) and generalized anxiety (37.3%) in the general Bangladeshi population. Generalized anxiety was observed more in females, those older than 30 years, who were married, had higher education and were non-governmental employees (13). Another study was conducted among healthcare workers working in a central public hospital that reported depression and anxiety and insomnia amongst 50 and 55% of the doctors, respectively (7).

Currently, there are very limited studies in Bangladesh assessing the factors associated with COVID-19 related distress, fear and coping strategies. In this study we aimed to assess the extent of psychological distress and the level of fear of COVID-19 among the Bangladeshi population and their coping strategies along with associated factors using previously validated tools. The high-risk groups of individuals identified through this study, could be targeted as the vulnerable groups who would require additional support for psychological well-being during the crisis period such as this pandemic.

## Methods

### Study Design and Settings

A cross-sectional study was conducted between August and September 2020, where participants from the community as well as clinical settings were approached via different online platforms.

### Study Population

People residing in Bangladesh (irrespective of nationality) during the study period, aged ≥18 years and capable of responding to an online questionnaire in English, were eligible for this study. Study participants consisted of general community members including COVID-19 patients, students, and healthcare professionals. If any study participant took <1 min to complete the questionnaire, he/she was excluded from the analyses due to unreliability of the responses.

### Sampling Technique and Sample Size

The Snowball sampling technique was used for collecting data. Once a participant filled up the online questionnaire, he/she was requested to forward the survey link to his/her personal /professional networks. The sampling technique was similar to our previous study, described elsewhere (10). The sample size was calculated using OpenEpi. Considering 164.71 million population of Bangladesh (14) assuming 50% prevalence of stress among the Bangladeshi (since no existing national data available on the prevalence of stress among Bangladeshis), at 95% confidence intervals and 80% power, the estimated minimum sample size was 385.

### Data Collection Tools and Technique

A structured survey questionnaire was developed using Google form. The survey was open as anyone having the survey link could participate in the study. All the contacts were made via online using emails, text messages and social media platforms such as Facebook, LinkedIn and Twitter. The survey was advertised via online platforms including emails, texts and social media. The emails and text messages were sent utilizing the professional and personal networks of the local study investigators, which included health professionals and students of the affiliated medical collages/hospitals. Besides personal social media platforms, the survey link was also shared to social media groups of general community people of Bangladesh. There were nine screens in total. The first screen of the online questionnaire contained the plain language statement and the consent form. The plain language statement mentioned about the aims of the study, types of data collected, anonymity of the responses collected, privacy and confidentiality of the collated data, data storage, details of investigators. On providing consent, participants could move to the next screen containing the screening questionnaire related to eligibility. If eligible, participants could proceed to filling out the full study questionnaire in the subsequent seven screens. No randomisation technique was applied for the questionnaire and adaptive questioning was used as applicable. The completeness of the questionnaire was indicated by the progress bar in the online questionnaire. There were also options of responding as “not applicable” or “no response”. Study participants had the options to go back and review/edit their responses accordingly.

Same questionnaire was used from the previous study conducted in Australia and Malaysia by the same research group (10). Psychological distress was measured using the Kessler Psychological Distress Scale (K-10), (15, 16) fear was measured using the Fear of COVID-19 Scale (FCV-19S), (17) and coping strategies were measured using Brief Resilient Coping Scale (BRCS) (18). The K-10 tool is a widely used psychometric tool, validated in different languages including English and used for public health research (15, 16) [the FCV-19S tool was recently developed in response to the COVID-19 pandemic, which has also been validated and used in many studies (17, 19, 20) validity and reliability have been tested for the BRCS tool in previous studies (18, 21, 22). Notably, they were recorded using a five-point Likert scale. There were two screening questions to determine eligibility to participate in the study, which was followed by a total of 39 questions. The details of each of the items are published in our previous study (10). A pre-test of the adapted version of the questionnaire was performed on a selective group of participants and the modification were completed before the data collection. Internal consistency of the questionnaire was satisfactory in the pilot study (data not shown). In addition, to minimize non-response bias, the following measures were adopted: the final questionnaire was pre-tested in both desktops/laptops/mobile phones/ipads so that the questionnaire appears correctly across all devices for the convenience of participants, a period of 2 months data collection period was ensured, survey reminders were sent to all potential participants across different networks at least three times within the data collection period. No incentive was provided to any study participant. In Bangladesh, essential service workers encompassed those individuals from essential workplaces including healthcare settings, pharmacies, food and groceries, schools and universities, public transports. Patients and/or the public were not involved in the design, conduct, reporting or dissemination plans of this research.

### Data Analyses

The database was downloaded from the Google platform and STATA v.12 was used for data analyses. Only completed questionnaire (*n* = 962) were analyzed. Descriptive analyses were followed by inferential analyses. Continuous variables were presented as means and standard deviations, and categorical variables were presented as proportions. Scoring in the K10 scale was re-defined into low (score 10–15) and moderate to very high (score 16–50), fear of COVID score was defined as BRCS categorized into low (score 4–13) and medium to high (score 14–20) resilient coping. Binary logistic regression was used to assess the association, results were presented as odds ratio (OR) and 95% confidence interval (CI). Multivariate analyses were conducted by adjusting for socio-demographic variables such as age, gender, living status, country of birth, education, and employment status. Then we reported adjusted OR (AOR) and 95% CI. Details of the analyses were discussed in the earlier study (10).

### Ethics

The study obtained approval from the Ethical Review Committee at Enam Medical College (Ref: EMC/ERC/2020/08-2). The survey was completely voluntary in nature and it was clarified in the plain language statement to explain it well, so that participants got the opportunity to have informed decision to participate in the study. No identifying information including any personal sensitive information were collected. Responses were anonymous and non-identifiable data were handled only by the investigators listed in the study.

## Results

A total of 1,016 people responded to the online survey, while 962 participants were included in this study (response rate was 95%). All respondents did not report the demographic information and responded to all questions, therefore, the total number of responses for each variable did not sum up to the total number of 962. Almost all of them were born in Bangladesh (98%), three-quarters of the study population were from the Dhaka division (*n* = 678, 73.1%) and lived in urban areas (*n* = 674, 72.6%) with the majority living with family members (814, 90%). Half of the participants (*n* = 478, 49.7%) belonged to the age group of 30–59 years and half were female (468, 50.4%). Almost two-thirds of the respondents had completed graduation (*n* = 624, 68%) and half of them had an income source during the pandemic (51%). While more than half of the participants reported that COVID-19 impacted their financial situation (64%), a little less than half reported moderate to great deal of perceived distress due to change in employment status (43%). Only a small proportion (7.5%) sought healthcare services to overcome COVID-related stress in the last 4 weeks. Frontline or essential service workers constituted half of the study population (*n* = 428, 48%) and over a quarter of the participants (*n* = 258, 28.2%) visited a healthcare provider in the last 4 weeks. Almost half of the respondents provided care to a family member/patient with known or suspected case of COVID-19 (52%). One in 10 participants (*n* = 95, 10.3%) reported having multiple co-morbidities and one in five reported having ever smoked (19%). Details of the characteristics of the study population are presented in Table 1.

**Table 1:** Characteristics of the study population.

**Characteristics**	**Total, *n* (%)**
Total study participants	962
Age (in years)	962
Mean (±SD)	32.2 (10.7)
Range	18 to 76
Age groups	962
18–29 years	453 (47.1)
30–59 years	478 (49.7)
≥60 years	31 (3.2)
Gender	928
Male	460 (49.6)
Female	468 (50.4)
Location in Bangladesh	928
Dhaka	678 (73.1)
Chottogram	59 (6.4)
Sylhet	18 (1.9)
Rajshahi	58 (6.3)
Rangpur	34 (3.7)
Khulna	40 (4.3)
Barisal	21 (2.3)
Myemensigh	20 (2.2)
Residence location in Bangladesh	928
Urban	674 (72.6)
Rural	254 (27.4)
Living status	908
Live without family members (on your own/shared house/others)	94 (10.4)
Live with family members (partner and/or children)	814 (89.6)
Born in Bangladesh	927
No	17 (1.8)
Yes	910 (98.2)
Completed level of education	923
Primary	25 (2.7)
Secondary	257 (27.8)
Trade/Certificate/Diploma	17 (1.8)
Degree (Bachelor)	322 (34.9)
Masters and above	302 (32.7)
Current employment condition	909
Unemployed/Housewife (No source of income)	334 (36.7)
Jobs affected by COVID-19 (lost job/working hours reduced/afraid of job loss)	109 (12.0)
Have an income source (employed/Government benefits)	466 (51.3)
Perceived distress due to change of employment status	889
A little to none	510 (57.4)
Moderate to a great deal	379 (42.6)
Self-identification as a frontline or essential service worker	892
No	464 (52.0)
Yes	428 (48.0)
COVID-19 impacted financial situation	928
No	339 (36.5)
Yes	589 (63.5)
Co-morbidities	922
No	599 (65.0)
Multiple co-morbidities	95 (10.3)
Hypertension	69 (7.5)
Psychiatric/Mental health problem	39 (4.2)
Cancer	37 (4.0)
Chronic respiratory diseases	28 (3.0)
High blood lipids	20 (2.2)
Diabetes/High blood sugar	18 (2.0)
Heart diseases	7 (0.8)
Chronic orthopedic problems	5 (0.5)
Stroke	1 (0.1)
Smoking	928
Never smoker	751 (80.9)
Ever smoker (Daily/Non-daily/Ex)	177 (19.1)
Increased smoking in the last 4 weeks	117
No	80 (68.40)
Yes	37 (31.6)
Current alcohol drinking (last 4 weeks)	918
No	888 (96.7)
Yes	30 (3.3)
Increased alcohol drinking over the last 4 weeks	26
No	19 (73.1)
Yes	7 (26.9)
Provided care to a family member/patient with known/suspected case of COVID-19	919
No	441 (48.0)
Yes	478 (52.0)
Experience related to COVID-19 pandemic (multiple responses possible)	886
No known exposure to COVID-19	635 (71.7)
I had recent overseas travel history and was in self-quarantine	27 (3.0)
I had been tested negative for COVID-19 but self-isolating	164 (18.5)
I had been tested positive for COVID-19	60 (6.8)
Self-identification as a patient (visited a healthcare provider in the last 4 weeks)	916
No	658 (71.8)
Yes	258 (28.2)
Healthcare service use in the last 4 weeks	361
Telehealth consultation/Use of national helpline	151 (41.8)
In-person visit to a healthcare provider	200 (55.4)
Used both services	10 (2.8)
Healthcare service use to overcome COVID-19 related stress in the last 4 weeks	915
No	846 (92.5)
Yes	69 (7.5)

More than two-thirds of the study participants (*n* = 530, 69.4%) experienced moderate to very high levels of psychological distress, and the mean (±SD) K-10 score was 21 (8.2) (Table 2). More than one-third of the participants (*n* = 357, 38.5%) reported high levels of fear of COVID-19, and the mean (±SD) FCV-19S score was 19.1 (7.3) (Table 3). More than half of the participants (*n* = 530, 57.1%) had medium to high resilient coping and the mean (±SD) BRCS score was 13.9 (3.2) (Table 4).

**Table 2:** Levels of psychological distress among the study participants.

**Anxiety and Depression Checklist (K10) (last 4 weeks)**	**Total**	**None, *n* (%)**	**A little, *n* (%)**	**Sometime, *n* (%)**	**Most of the time, *n* (%)**	**All the time, *n* (%)**
1. About how often did you feel tired out for no good reason?	928	217 (23.4)	179 (19.3)	377 (40.6)	137 (14.8)	18 (1.9)
2. About how often did you feel nervous?	928	232 (25.0)	259 (27.9)	332 (35.8)	91 (9.8)	14 (1.5)
3. About how often did you feel so nervous that nothing could calm you down?	928	492 (53.0)	217 (23.4)	174 (18.8)	42 (4.5)	3 (0.3)
4. About how often did you feel hopeless?	928	337 (36.3)	232 (25.0)	226 (24.4)	110 (11.9)	23 (2.5)
5. About how often did you feel restless or fidgety?	928	379 (40.8)	238 (25.6)	220 (23.7)	80 (8.6)	11 (1.2)
6. About how often did you feel so restless you could not sit still?	928	562 (60.6)	203 (21.9)	124 (13.4)	34 (3.7)	5 (0.5)
7. About how often did you feel so depressed?	928	304 (32.8)	227 (24.5)	268 (28.9)	104 (11.2)	25 (2.6)
8. About how often did you feel that everything was an effort?	928	308 (33.2)	243 (26.2)	246 (26.5)	105 (11.3)	26 (2.8)
9. About how often did you feel so sad that nothing could cheer you up?	928	392 (42.2)	226 (24.4)	217 (23.4)	77 (8.0)	16 (1.7)
10. About how often did you feel worthless?	928	446 (48.1)	218 (23.5)	174 (18.8)	64 (6.9)	26 (2.8)
K10 score (total)	928					
Mean (±SD)	21.0 (8.2)					
Range	10 to 50					
Level of psychological distress (K10 categories)	928					
Low (score 10–15)	284 (30.6)					
Moderate (score 16–21)	243 (26.2)					
High (score 22–29)	246 (26.5)					
Very high (score 30–50)	155 (16.7)					

**Table 3:** Levels of fear of COVID-19 among the study participants.

**Fear of COVID-19 Scale (FCV-19S) Individual Items**	**Total**	**Strongly disagree, *n* (%)**	**Somewhat disagree, *n* (%)**	**Neither agree nor disagree, *n* (%)**	**Somewhat agree, *n* (%)**	**Strongly agree, *n* (%)**
1. I am most afraid of COVID-19	928	167 (18.0)	109 (11.7)	203 (21.9)	306 (33.0)	143 (15.4)
2. It makes me uncomfortable to think about COVID-19	928	161 (17.3)	114 (12.3)	161 (17.3)	347 (37.4)	145 (15.6)
3. My hands become clammy when I think about COVID-19	928	393 (42.3)	166 (17.9)	196 (21.1)	120 (12.9)	53 (5.7)
4. I am afraid of losing my life because of COVID-19	928	261 (28.1)	123 (13.3)	186 (20.0)	251 (27.0)	107 (11.5)
5. When watching news and stories about COVID-19 on social media, I become nervous or anxious	928	177 (19.1)	98 (10.6)	148 (15.9)	355 (38.3)	150 (16.2)
6. I cannot sleep because I'm worrying about getting COVID-19	928	461 (49.7)	134 (14.4)	166 (17.9)	122 (13.1)	45 (4.8)
7. My heart races or palpitates when I think about getting COVID-19	928	378 (40.7)	130 (14.0)	158 (17.0)	190 (20.5)	72 (7.8)
FCV-19S score (total)	928					
Mean (±SD)	19.1 (7.3)					
Range	7 to 35					
Level of fear of COVID-19 (FCV-19S categories)	928					
Low (score 7–21)	571 (61.5)					
High (score 22–35)	357 (38.5)					

**Table 4:** Coping during COVID-19 pandemic among the study participants.

**Brief Resilient Coping Scale (BRCS) Individual Items**	**Total**	**Does not describe me at all, *n* (%)**	**Does not describe me, *n* (%)**	**Neutral, *n* (%)**	**Describes me, *n* (%)**	**Describes me very well, *n* (%)**
1. I look for creative ways to alter difficult situations	928	57 (6.1)	80 (8.6)	407 (43.9)	267 (28.8)	117 (12.6)
2. Regardless of what happens to me, I believe I can control my reaction to it	928	45 (4.8)	90 (9.7)	321 (34.6)	340 (36.6)	132 (14.2)
3. I believe I can grow in positive ways by dealing with difficult situations	928	38 (4.1)	59 (6.4)	265 (28.6)	395 (42.6)	171 (18.4)
4. I actively look for ways to replace the losses I encounter in life	928	38 (4.1)	75 (8.1)	337 (36.3)	350 (37.7)	128 (13.8)
BRCS score (total)	928					
Mean (±SD)	13.9 (3.2)					
Range	4 to 20					
Level of coping (BRCS categories)	928					
Low resilient copers (score 4–13)	398 (42.9)					
Medium resilient copers (score 14–16)	366 (39.4)					
High resilient copers (score 17–20)	164 (17.7)					

### Psychological Distress

Table 5 shows unadjusted and adjusted analyses for identifying factors associated with moderate to very high psychological distress. Following adjustment of potential confounders, higher levels of psychological distress were found to be associated with being a female, born in Bangladesh, having a graduate or above level of education. A range of other factors were also associated with higher distress, such as having moderate to a great deal of perceived distress, including change in employment, being a frontline/essential service worker, impacted financial situation due to COVID-19, having psychiatric/mental health problems, having multiple co-morbidities, being a smoker (at any time), providing care to a known/suspected case of COVID-19, having a recent overseas travel history and being in self-quarantine, having negative test results for COVID-19 but being in self-isolation, having positive test results for COVID-19, being a patient, and having higher levels of fear of COVID-19. In contrast, living in a rural area and having an income source during the pandemic were associated with lower levels of psychological distress following adjustment of potential confounders.

**Table 5:** Factors associated with moderate to very high psychological distress among the study participants (based on K10 score).

**Characteristics**	**Moderate to very high (score 16–50), *n* (%)**	**Low (score 10–15), *n* (%)**	**Unadjusted analyses**			**Adjusted analyses**		
			***p***	**OR**	**95% CI**	***p***	**AOR**	**95% CI**
Total study participants	644	284						
Age groups	644	284						
18–29 years	317 (49.2)	119 (41.9)		1			1	
30–59 years	306 (47.5)	156 (54.9)	***0.036***	***0.74***	***0.55–0.98***	0.295	0.85	0.62–1.16
≥60 years	21 (3.3)	9 (3.2)	0.748	0.88	0.39–1.97	0.657	0.82	0.35–1.94
Gender	644	284						
Male	284 (44.1)	176 (62.0)		1			1	
Female	360 (55.9)	108 (38.0)	***0.000***	***2.07***	***1.55–2.75***	***0.000***	***1.81***	***1.33–2.47***
Living status	634	274						
Live without family members (on your own/shared house/others)	60 (9.5)	34 (12.4)		1			1	
Live with family members (partner and/or children)	574 (90.5)	240 (87.6)	0.181	1.36	0.87–2.12	0.248	1.33	0.82–2.16
Residence location in Bangladesh	644	284						
Urban	506 (78.6)	168 (59.2)		1			1	
Rural	138 (21.4)	116 (40.8)	***0.000***	***0.40***	***0.29–0.53***	***0.000***	***0.38***	***0.27–0.54***
Born in Bangladesh	643	284						
No	8 (1.2)	9 (3.2)		1			1	
Yes	635 (98.8)	275 (96.8)	***0.044***	***2.60***	***0.99–6.80***	***0.012***	***3.74***	***1.34–10.5***
Completed level of education	639	284						
Primary	11 (1.7)	14 (4.9)		1			1	
Secondary	166 (26.0)	91 (32.0)	***0.047***	***2.32***	***1.01–5.32***	0.158	1.92	0.78–4.72
Trade/Certificate/Diploma	9 (1.4)	8 (2.8)	0.570	1.43	0.42–4.93	0.666	1.34	0.35–5.07
Degree (Bachelor)	239 (37.4)	83 (29.2)	***0.002***	***3.66***	***1.60–8.39***	***0.008***	***3.52***	***1.38–8.96***
Masters and above	214 (33.5)	88 (31.0)	***0.007***	***3.10***	***1.35–7.08***	***0.045***	***2.62***	***1.02–6.70***
Current employment condition	631	278						
Unemployed/Home duties	234 (37.1)	100 (36.0)		1			1	
Jobs affected by COVID-19 (lost job/working hours reduced/afraid of job loss)	71 (11.3)	38 (13.7)	0.336	0.80	0.50–1.26	0.306	0.75	0.44–1.30
Have an income source (employed/Government benefits)	326 (51.7)	140 (50.4)	0.975	1.00	0.73–1.35	***0.015***	***0.59***	***0.38–0.90***
Perceived distress due to change of employment status	615	274						
A little to none	320 (52.0)	190 (69.3)		1			1	
Moderate to a great deal	295 (48.0)	84 (30.7)	***0.000***	***2.09***	***1.54–2.82***	***0.000***	***2.32***	***1.63–3.29***
Self-identification as a frontline or essential service worker	621	271						
No	304 (49.0)	160 (59.0)		1			1	
Yes	317 (51.0)	111 (41.0)	***0.006***	***1.50***	***1.13–2.01***	***0.030***	***1.50***	***1.04–2.15***
COVID-19 impacted financial situation	644	284						
No	215 (33.4)	124 (43.7)		1			1	
Yes	429 (66.6)	160 (56.3)	***0.003***	***1.55***	***1.16–2.06***	***0.000***	***2.05***	***1.49–2.84***
Co-morbidities	638	284						
No	395 (61.9)	204 (71.8)		1			1	
Psychiatric/Mental health problem	36 (5.6)	3 (1.1)	***0.003***	***6.20***	***1.89–20.4***	***0.025***	***4.03***	***1.19–13.7***
Other co-morbidities^*^	132 (20.7)	57 (20.1)	0.321	1.20	0.84–1.70	0.062	1.46	0.98–2.16
Multiple co-morbidities	75 (11.8)	20 (7.0)	***0.013***	***1.94***	***1.15–3.26***	***0.007***	***2.17***	***1.24–3.80***
Smoking	644	284						
Never smoker	513 (79.7)	238 (83.8)		1			1	
Ever smoker (Daily/Non-daily/Ex)	131 (20.3)	46 (16.2)	0.139	1.32	0.91–1.91	***0.001***	***2.02***	***1.32–3.09***
Increased smoking in the last 4 weeks	94	23						
No	61 (64.9)	19 (82.6)		1			1	
Yes	33 (35.1)	4 (17.4)	0.101	2.57	0.81–8.18	0.170	2.51	0.67–9.32
Current alcohol drinking (last 4 weeks)	637	281						
No	614 (96.4)	274 (97.5)		1			1	
Yes	23 (3.6)	7 (2.5)	0.379	1.47	0.62–3.46	0.279	1.67	0.66–4.22
Increased alcohol drinking over the last 4 weeks	19	7						
No	13 (68.4)	6 (85.7)		1			1	
Yes	6 (31.6)	1 (14.3)	0.378	2.77	0.27–28.4	0.389	12.6	0.04–4056
Provided care to a family member/patient with known/suspected case of COVID-19	640	279						
No	272 (42.5)	169 (60.6)		1			1	
Yes	368 (57.5)	110 (39.4)	***0.000***	***2.08***	***1.56–2.77***	***0.000***	***1.96***	***1.40–2.72***
Experience related to COVID-19 pandemic	611	275						
No known exposure to COVID-19	408 (66.8)	227 (82.5)		1			1	
I had recent overseas travel history and was in self-quarantine	23 (3.8)	4 (1.5)	***0.034***	***3.20***	***1.09–9.37***	***0.024***	***4.59***	***1.23–17.2***
I had been tested negative for COVID-19 but self-isolating	130 (21.3)	34 (12.4)	***0.000***	***2.12***	***1.41–3.21***	***0.000***	***2.63***	***1.68–4.13***
I had been tested positive for COVID-19	50 (8.2)	10 (3.6)	***0.004***	***2.78***	***1.38–5.59***	***0.015***	***2.53***	***1.19–5.34***
Self-identification as a patient (visited a healthcare provider in the last 4 weeks)	636	280						
No	432 (67.9)	226 (80.7)		1			1	
Yes	204 (32.1)	54 (19.3)	***0.000***	***1.98***	***1.41–2.78***	***0.000***	***2.26***	***1.56–3.27***
Healthcare service use in the last 4 weeks	275	86						
Telehealth consultation/Use of national helpline	120 (43.6)	31 (36.0)		1			1	
In-person visit to a healthcare provider	148 (53.8)	52 (60.5)	0.233	0.74	0.44–1.22	0.317	1.35	0.75–2.41
Used both services	7 (2.5)	3 (3.5)	0.481	0.60	0.15–2.47	0.374	0.49	0.10–2.39
Level of fear of COVID-19 (FCV-19S categories)	644	284						
Low (score 7–21)	345 (53.6)	226 (79.6)		1			1	
High (score 22–35)	299 (46.4)	58 (20.4)	***0.000***	***3.38***	***2.43–4.69***	***0.000***	***3.27***	***2.29–4.66***
Level of coping (BRCS categories)	644	284						
Low resilient coping (score 4–13)	281 (43.6)	117 (41.2)		1			1	
Medium to high resilient coping (score 14−20)	363 (56.4)	167 (58.8)	0.490	0.91	0.68–1.20	0.178	0.81	0.59–1.10
Healthcare service use to overcome COVID-19 related stress in the last 4 weeks	638	277						
No	585 (91.7)	261 (94.2)		1			1	
Yes	53 (8.3)	16 (5.8)	0.183	1.48	0.83-2.63	0.199	1.53	0.80-2.92

*Adjusted for: age, gender, living status, residence location, born in Bangladesh, education and employment*.

* *Cardiac disases/Stroke/Hypertension/Hyperlipidemia/Diabetes/Cancer/Chronic respiratory illness*.

*Significant results are indicated as bold and italic*.

### Fear of COVID-19

Higher levels of fear of COVID-19 were associated with several factors following adjustment of potential confounders, such as living with family members, being born in Bangladesh, having Bachelors and Masters level of education or above, having moderate to a great deal of perceived distress due to changes in employment, impacted financial situation due to COVID-19, having multiple co-morbidities, being a patient, and having moderate to very high level of psychological distress (Table 6).

**Table 6:** Factors associated with high levels of fear of COVID-19 among the study participants (based on FCV-19S score).

**Characteristics**	**High (score 22–35), *n* (%)**	**Low (score 7–21), *n* (%)**	**Unadjusted analyses**			**Adjusted analyses**		
			***p***	**OR**	**95% CI**	***p***	**AOR**	**95% CI**
Total study participants	357	571						
Age groups	357	571						
18–29 years	174 (48.7)	262 (45.9)		1			1	
30–59 years	172 (48.2)	290 (50.8)	0.410	0.89	0.68–1.17	0.952	1.01	0.76–1.34
≥60 years	11 (3.1)	19 (3.3)	0.726	0.87	0.40–1.88	0.691	1.18	0.52–2.65
Gender	357	571						
Male	165 (46.2)	295 (51.7)		1			1	
Female	192 (53.8)	276 (48.3)	0.106	1.24	0.95–1.62	0.265	1.18	0.88–1.56
Living status	352	556						
Live without family members (on your own/shared house/others)	26 (7.4)	68 (12.2)		1			1	
Live with family members (partner and/or children)	326 (92.6)	488 (87.8)	***0.020***	***1.75***	***1.09***–***2.80***	***0.028***	***1.74***	***1.06***–***2.87***
Residence location in Bangladesh	357	571						
Urban	275 (77.0)	399 (69.9)		1			1	
Rural	82 (23.0)	172 (30.1)	***0.017***	***0.69***	***0.51***–***0.94***	0.321	0.84	0.60–1.18
Born in Bangladesh	357	570						
No	2 (0.6)	15 (2.6)		1			1	
Yes	355 (99.4)	555 (97.4)	***0.022***	***4.80***	***1.09***–***21.1***	***0.037***	***4.98***	***1.10***–***22.4***
Completed level of education	353	570						
Primary	4 (1.1)	21 (3.7)		1			1	
Secondary	82 (23.2)	175 (30.7)	0.109	2.46	0.82–7.40	0.167	2.21	0.72–6.78
Trade/Certificate/Diploma	2 (0.6)	15 (2.6)	0.701	0.70	0.11–4.33	0.704	0.70	0.11–4.44
Degree (Bachelor)	128 (36.3)	194 (34.0)	***0.026***	***3.46***	***1.16***–***10.3***	***0.020***	***3.83***	***1.23***–***11.9***
Masters and above	137 (38.8)	165 (28.9)	***0.008***	***4.36***	***1.46***–***13.0***	***0.012***	***4.32***	***1.38***–***13.5***
Current employment condition	349	560						
Unemployed/Home duties	122 (35.0)	212 (37.9)		1			1	
Jobs affected by COVID-19 (lost job/working hours reduced/afraid of job loss)	39 (11.2)	70 (12.5)	0.888	0.97	0.62–1.52	0.252	0.74	0.45–1.23
Have an income source (employed/Government benefits)	188 (53.9)	278 (49.6)	0.275	1.18	0.88–1.57	0.280	0.81	0.56–1.18
Perceived distress due to change of employment status	342	547						
A little to none	174 (50.9)	336 (61.4)		1			1	
Moderate to a great deal	168 (49.1)	211 (38.6)	***0.002***	***1.54***	***1.17***–***2.02***	***0.003***	***1.56***	***1.16***–***2.11***
Self-identification as a frontline or essential service worker	343	549						
No	165 (48.1)	299 (54.5)		1			1	
Yes	178 (51.9)	250 (45.5)	0.064	1.29	0.99–1.69	0.118	1.30	0.94–1.81
COVID-19 impacted financial situation	357	571						
No	112 (31.4)	227 (39.8)		1			1	
Yes	245 (68.6)	344 (60.2)	***0.010***	***1.44***	***1.09***–***1.91***	***0.000***	***1.78***	***1.31***–***2.40***
Co-morbidities	355	567						
No	212 (59.7)	387 (68.3)		1			1	
Psychiatric/Mental health problem	17 (4.8)	22 (3.9)	0.303	1.41	0.73–2.71	0.173	1.65	0.80–3.40
Other co-morbidities^*^	76 (21.4)	113 (19.9)	0.231	1.23	0.88–1.72	0.156	1.30	0.91–1.86
Multiple co-morbidities	50 (14.1)	45 (7.9)	***0.001***	***2.03***	***1.31***–***3.14***	***0.002***	***2.04***	***1.29***–***3.24***
Smoking	357	571						
Never smoker	291 (81.5)	460 (80.6)		1			1	
Ever smoker (Daily/Non-daily/Ex)	66 (18.5)	111 (19.4)	0.719	0.94	0.67–1.32	0.716	1.07	0.73–1.58
Increased smoking in the last 4 weeks	46	71						
No	28 (60.9)	52 (73.2)		1			1	
Yes	18 (39.1)	19 (26.8)	0.160	1.76	0.80–3.88	0.067	2.38	0.94–6.00
Current alcohol drinking (last 4 weeks)	352	566						
No	342 (97.2)	546 (96.5)		1			1	
Yes	10 (2.8)	20 (3.5)	0.566	0.80	0.37–1.73	0.674	1.20	0.52–2.75
Increased alcohol drinking over the last 4 weeks	8	18						
No	7 (87.5)	12 (66.7)		1			1	
Yes	1 (12.5)	6 (33.3)	0.269	0.29	0.03–2.89	0.706	0.45	0.01–27.5
Provided care to a family member/patient with known/suspected case of COVID-19	354	565						
No	163 (46.0)	278 (49.2)		1			1	
Yes	191 (54.0)	287 (50.8)	0.351	1.14	0.87–1.48	0.836	0.97	0.72–1.31
Experience related to COVID-19 pandemic	343	543						
No known exposure to COVID-19	240 (70.0)	395 (72.7)		1			1	
I had recent overseas travel history and was in self-quarantine	13 (3.8)	14 (2.6)	0.281	1.53	0.71–3.31	0.102	2.03	0.87–4.76
I had been tested negative for COVID-19 but self-isolating	64 (18.7)	100 (18.4)	0.773	1.05	0.74–1.50	0.506	1.13	0.78–1.65
I had been tested positive for COVID-19	26 (7.6)	34 (6.3)	0.400	1.26	0.74–2.15	0.534	1.20	0.67–2.16
Self-identification as a patient (visited a healthcare provider in the last 4 weeks)	350	566						
No	237 (67.7)	421 (74.4)		1			1	
Yes	113 (32.3)	145 (25.6)	***0.029***	***1.38***	***1.03***–***1.86***	***0.003***	***1.60***	***1.18***–***2.18***
Healthcare service use in the last 4 weeks	159	202						
Telehealth consultation/Use of national helpline	69 (43.4)	82 (40.6)		1			1	
In-person visit to a healthcare provider	85 (53.5)	115 (56.9)	0.550	0.88	0.57–1.34	0.590	1.14	0.72–1.80
Used both services	5 (3.1)	5 (2.5)	0.792	1.19	0.33–4.28	0.410	1.83	0.44–7.68
Level of psychological distress (K10 categories)	357	571						
Low (score 10–15)	58 (16.2)	226 (39.6)		1			1	
Moderate to Very High (score 16–50)	299 (83.8)	345 (60.4)	***0.000***	***3.38***	***2.43***–***4.69***	***0.000***	***3.29***	***2.31***–***4.69***
Level of coping (BRCS categories)	357	571						
Low resilient coping (score 4–13)	156 (43.7)	242 (42.4)		1			1	
Medium to high resilient coping (score 14–20)	201 (56.3)	329 (57.6)	0.694	0.95	0.73–1.24	0.345	0.87	0.66–1.16
Healthcare service use to overcome COVID-19 related stress in the last 4 weeks	353	562						
No	320 (90.7)	526 (93.6)		1			1	
Yes	33 (9.3)	36 (6.4)	0.101	1.51	0.92–2.47	0.057	1.68	0.99–2.87

*Adjusted for: age, gender, living status, residence location, born in Bangladesh, education and employment*.

* *Cardiac disases/Stroke/Hypertension/Hyperlipidemia/Diabetes/Cancer/Chronic respiratory illness*.

*Significant results are indicated as bold and italic*.

### Coping Strategies

Multivariate analyses showed that study participants who had an income source had medium to high resilient coping, whereas those with pre-existing psychiatric/mental health problems had low resilient coping (Table 7).

**Table 7:** Factors associated with medium to high resilience coping among the study participants (based on BRCS score).

**Characteristics**	**Medium to High (score 14–20), *n* (%)**	**Low (score 4–13), *n* (%)**	**Unadjusted analyses**			**Adjusted analyses**		
			***p***	**OR**	**95% CI**	***p***	**AOR**	**95% CI**
Total study participants	530	398						
Age groups	530	398						
18**–**29 years	237 (44.7)	199 (50.0)		1			1	
30**–**59 years	275 (51.9)	187 (47.0)	0.118	1.23	0.95**–**1.61	0.191	1.21	0.91**–**1.60
≥60 years	18 (3.4)	12 (3.0)	0.549	1.26	0.59**–**2.68	0.381	1.43	0.64**–**3.18
Gender	530	398						
Male	257 (48.5)	203 (51.0)		1			1	
Female	273 (51.5)	195 (49.0)	0.448	1.11	0.85–1.43	0.791	1.04	0.79–1.37
Living status	524	384						
Live without family members (on your own/shared house/others)	46 (8.8)	48 (12.5)		1			1	
Live with family members (partner and/or children)	478 (91.2)	336 (87.5)	0.069	1.48	0.97–2.28	0.093	1.47	0.94–2.31
Residence location in Bangladesh	530	398						
Urban	396 (74.7)	278 (69.8)		1			1	
Rural	134 (25.3)	120 (30.2)	0.100	0.78	0.59–1.05	0.145	0.78	0.57–1.09
Born in Bangladesh	529	398						
No	8 (1.5)	9 (2.3)		1			1	
Yes	521 (98.5)	389 (97.7)	0.400	1.51	0.58–3.94	0.533	1.38	0.50–3.76
Completed level of education	526	397						
Primary	13 (2.5)	12 (3.0)		1			1	
Secondary	155 (29.5)	102 (25.7)	0.421	1.40	0.62–3.20	0.623	1.24	0.52–2.93
Trade/Certificate/Diploma	10 (1.9)	7 (1.8)	0.663	1.32	0.38–4.58	0.793	0.84	0.23–3.06
Degree (Bachelor)	166 (31.6)	156 (39.3)	0.966	0.98	0.44–2.22	0.540	0.76	0.32–1.83
Masters and above	182 (34.6)	120 (30.2)	0.420	1.40	0.62–3.17	0.987	0.99	0.41–2.41
Current employment condition	520	389						
Unemployed/Home duties	182 (35.0)	152 (39.1)		1			1	
Jobs affected by COVID-19 (lost job/working hours reduced/afraid of job loss)	57 (11.0)	52 (13.4)	0.689	0.92	0.59–1.41	0.935	0.98	0.61–1.59
Have an income source (employed/Government benefits)	281 (54.0)	185 (47.6)	0.101	1.27	0.95–1.69	***0.041***	***1.46***	***1.02–2.11***
Perceived distress due to change of employment status	512	377						
A little to none	309 (60.4)	201 (53.3)		1			1	
Moderate to a great deal	203 (39.6)	176 (46.7)	***0.036***	***0.75***	***0.57–0.98***	0.094	0.78	0.58–1.04
Self-identification as a frontline or essential service worker	513	379						
No	279 (54.4)	185 (48.8)		1			1	
Yes	234 (45.6)	194 (51.2)	0.100	0.80	0.61–1.04	0.047	0.72	0.52–1.00
COVID-19 impacted financial situation	530	398						
No	202 (38.1)	137 (34.4)		1			1	
Yes	328 (61.9)	261 (65.6)	0.248	0.85	0.65–1.12	0.554	0.92	0.69–1.22
Co-morbidities	528	394						
No	349 (66.1)	250 (63.5)		1			1	
Psychiatric/Mental health problem	12 (2.3)	27 (6.9)	***0.001***	***0.32***	***0.16–0.64***	***0.001***	***0.25***	***0.11–0.54***
Other co-morbidities^*^	114 (21.6)	75 (19.0)	0.617	1.09	0.78–1.52	0.603	1.10	0.77–1.56
Multiple co-morbidities	53 (10.0)	42 (10.7)	0.650	0.90	0.58–1.40	0.405	0.82	0.52–1.30
Smoking	530	398						
Never smoker	427 (80.6)	324 (81.4)		1			1	
Ever smoker (Daily/Non-daily/Ex)	103 (19.4)	74 (18.6)	0.747	1.06	0.76–1.47	0.358	1.19	0.82–1.74
Increased smoking in the last 4 weeks	67	50						
No	46 (68.7)	34 (68.0)		1			1	
Yes	21 (31.3)	16 (32.0)	0.940	0.97	0.44–2.13	0.781	1.15	0.43–3.05
Current alcohol drinking (last 4 weeks)	526	392						
No	513 (97.5)	375 (95.7)		1			1	
Yes	13 (2.5)	17 (4.3)	0.116	0.56	0.27–1.17	0.400	0.71	0.33–1.56
Increased alcohol drinking over the last 4 weeks	12	14						
No	10 (83.3)	9 (64.3)		1			1	
Yes	2 (16.7)	5 (35.7)	0.275	0.36	0.06–2.34	0.363	0.17	0.00–7.52
Provided care to a family member/patient with known/suspected case of COVID-19	528	391						
No	252 (47.7)	189 (48.3)		1			1	
Yes	276 (52.3)	202 (51.7)	0.855	1.03	0.79–1.33	0.962	1.01	0.75–1.35
Experience related to COVID-19 pandemic	507	379						
No known exposure to COVID-19	347 (68.4)	288 (76.0)		1			1	
I had recent overseas travel history and was in self-quarantine	18 (3.6)	9 (2.4)	0.223	1.66	0.73–3.75	0.080	2.25	0.91–5.56
I had been tested negative for COVID-19 but self-isolating	101 (19.9)	63 (16.6)	0.111	1.33	0.94–1.89	0.124	1.33	0.92–1.93
I had been tested positive for COVID-19	41 (8.1)	19 (5.0)	***0.044***	***1.79***	***1.02–3.15***	0.089	1.70	0.92–3.14
Self-identification as a patient (visited a healthcare provider in the last 4 weeks)	527	389						
No	371 (70.4)	287 (73.8)		1			1	
Yes	156 (29.6)	102 (26.2)	0.261	1.18	0.88–1.59	0.317	1.17	0.86–1.58
Healthcare service use in the last 4 weeks	207	154						
Telehealth consultation/Use of national helpline	93 (44.9)	58 (37.7)		1			1	
In-person visit to a healthcare provider	111 (53.6)	89 (57.8)	0.253	0.78	0.51–1.20	0.487	0.84	0.52–1.36
Used both services	3 (1.4)	7 (4.5)	0.063	0.27	0.07–1.07	0.160	0.34	0.07–1.54
Level of psychological distress (K10 categories)	530	398						
Low (score 10**–**15)	167 (31.5)	117 (29.4)		1			1	
Moderate to Very High (score 16–50)	363 (68.5)	281 (70.6)	0.490	0.91	0.68–1.20	0.162	0.80	0.59–1.09
Level of fear of COVID-19 (FCV-19S categories)	530	398						
Low (score 7–21)	329 (62.1)	242 (60.8)		1			1	
High (score 22–35)	201 (37.9)	156 (39.2)	0.694	0.95	0.73–1.24	0.369	0.88	0.66–1.17
Healthcare service use to overcome COVID-19 related stress in the last 4 weeks	524	391						
No	484 (92.4)	362 (92.6)		1			1	
Yes	40 (7.6)	29 (7.4)	0.902	1.03	0.63–1.70	0.770	1.08	0.63–1.85

*Adjusted for: age, gender, living status, residence location, born in Bangladesh, education and employment*.

* *Cardiac disases/Stroke/Hypertension/Hyperlipidemia/Diabetes/Cancer/Chronic respiratory illness*.

*Significant results are indicated as bold and italic*.

## Discussion

This is one of the first studies carried out among Bangladeshi residents using validated tools to assess the extent and factors associated with psychological distress, level of fear, and coping strategies during the COVID-19 pandemic. Several factors were identified that were associated with a higher level of psychological distress and fear. Being born in Bangladesh, having completed graduate level education and above, perceived distress due to change of employment status, COVID-19 impacting financial situation, having multiple co-morbidities and visiting a health care provider in the past 4 weeks was associated with higher levels of psychological distress and fear. Being a female, being a frontline or essential service worker, having pre-existing mental health problems, being an ever smoker, providing care to a known/suspected case of COVID-19, having an overseas travel history, being in quarantine, having negative test results for COVID-19 but being in self-isolation, having positive test results for COVID-19, and having higher levels of fear of COVID-19 was associated with higher psychological distress. In contrast, living in rural areas and having an income source was associated with lower psychological distress. Living with family members and increased psychological distress was associated with a higher level of fear. People who had an income source during the pandemic were more likely to be resilient. However, people with pre-existing mental health conditions were less likely to be resilient.

Around 69% of respondents experienced moderate to high levels of psychological distress in our study which was similar to the findings from a study of psychological distress during the pandemic in Victoria, Australia (63%) (10) and slightly higher than seen in a nationwide study covering 193 cities in China where 53.8% of participants reported having moderate to severe psychological distress during the pandemic (9, 12). The study finding was also much higher than that found in a national survey (conducted during the non-pandemic period) in Bangladesh where prevalence of mental disorder was between 6.5 and 31.0% in adults (23) and another large-scale nationwide survey across 64 districts in Bangladesh showed the prevalence of mental illness as around 33% (24). However, the latter survey was done when COVID 19 pandemic was mostly confined to the imported transmission and no data regarding community spread was reported in Bangladesh. The Australian study also had a good representation of overseas respondents which could explain the similar prevalence of psychological distress in the two studies (10).

Similar to the Australian study, this study also found females and those with mental health problems to be at risk of experiencing higher psychological distress (10). A high level of mental illness was observed in a study among the US people during the 1st month of infection particularly among female, Hispanic ethnicity, and those with diagnosed previous mental illness (25). Studies have shown that people with pre-existing mental health disorders including anxiety disorders, existing health anxiety (those who worry excessively about having or contracting illnesses), and depression and post-traumatic stress are at increased risk of higher anxiety during the COVID-19 outbreak (10, 26). Our study also showed a similarity with significantly higher levels of psychological distress among participants having pre-existing comorbidities such as psychiatric or mental health issues. Individuals who were self-isolating or quarantining also showed a high level of psychological distress, similar to previous findings (27). A sense of stigma from other family members or friends might have contributed to such high levels of distress (28).^.^Similar to previous studies, frontline healthcare workers or essential service holders showed a higher level of distress during the pandemic due to increased work-load, infection of colleagues, death of young professionals, infection of family members, lack of protective measures, and an increase in the frequency of medical violence (29, 30).

A major adverse consequence of the COVID-19 pandemic is likely to increase social isolation and loneliness which are strongly associated with psychological distress as found in our study. Tracking loneliness and intervening early are important public health priorities. Social isolation and loneliness are distinct and might represent different risk pathways. A higher level of distress was observed in females which was consistent with results from Bangladesh (24), Australia (10), China (31) and the USA (25). This can be postulated due to the effects of long-term stay at home, increased domestic violence (32) and being a focal person as a primary caregiver for the affected person at home (33).

The results of our study illustrated additional aspects of fear and distress. No association was found between fear and having any existing comorbidities or increased healthcare utilization, providing care to family or patients with a known and suspected case of COVID-19. These could be explained as the survey was administered during August 2020 when the number of cases declined as per previous data and the sense of catastrophic nature of the pandemic was not pronounced. The majority of our respondents were essential service workers or frontline workers who already adapted to the situation.

One of the major strengths of this study was the use of validated tools to investigate the factors associated with psychological distress, fear and coping strategies among a large number of the Bangladeshi population during the COVID-19 pandemic. The study, however, was not without limitations. As this study was an online survey, response wise preponderance of the younger people was noted as they were presumably more active on social media and had more online access. The study was conducted in English, so those who were not well-versed in English were not able to take part in the study. Use of snowball sampling technique potentially introduced selection bias and the self-reporting nature of the survey could also lead to reporting bias. However, due to the nationwide restrictions of movement, such sampling method was deemed feasible at that pandemic period. The survey responses in this study were predominantly from Dhaka division, although the survey link was shared all over Bangladesh through various social media platforms and emails. An important limitation from our study is, participants who might have tested positive to COVID-19 or those whose family members have tested positive with COVID-19 infection were likely to have reported more depressive symptoms than those who had not. Therefore, the present findings cannot be generalized to the healthy Bangladeshi population. In addition, we do acknowledge that we might have missed more marginalized or vulnerable group of population in our study (e.g., more isolated, experiencing violence or exploitation, in more intensive or less flexible employment or caring roles, or migrant or other minority status); therefore, our findings were potentially underestimated compared to the actual situations there. Due to the exploratory nature of the study, some of the significant findings could be due to chance (using a significance level of 0.05).

## Conclusion

The study shows a high level of psychological distress and fear among Bangladeshi people during the COVID-19 pandemic. People with pre-existing mental illness, females, and frontline workers require special attention as they are most affected by the pandemic leading to increased psychological distress. We have used K-10 and BRCS to assess psychological distress and coping in our study. However, there are other assessment tools to measure those issues, future studies can examine the difference in measurement by using more than one study tool. The risk factors identified in this study will help in designing target-based screening for the at-risk people to reduce the burden of mental health in the society.

## Data Availability Statement

The original contributions generated for this study are included in the article/supplementary material, further inquiries can be directed to the corresponding author/s.

## Ethics Statement

The studies involving human participants were reviewed and approved by Ethical Review Committee at Enam Medical College (Ref: EMC/ERC/2020/08-2).

## Author Contributions

MAR conceived the study and performed the statistical analyses. TB, SR, ASBM, SMA, FS, MS, SMSI, WC, and MAR contributed for planning the study. TB, SR, SMA, FS, and MS drafted the manuscript. TB, SR, AW, SMYA, ZZC, BMMU, and MMR supported data collection activities at local settings. WC and MAR provided critical review of the manuscript. All authors read and approved the final manuscript.

## Conflict of Interest

The authors declare that the research was conducted in the absence of any commercial or financial relationships that could be construed as a potential conflict of interest.

## Publisher's Note

All claims expressed in this article are solely those of the authors and do not necessarily represent those of their affiliated organizations, or those of the publisher, the editors and the reviewers. Any product that may be evaluated in this article, or claim that may be made by its manufacturer, is not guaranteed or endorsed by the publisher.
